# SOX2 protein biochemistry in stemness, reprogramming, and cancer: the PI3K/AKT/SOX2 axis and beyond

**DOI:** 10.1038/s41388-019-0997-x

**Published:** 2019-09-02

**Authors:** Thorsten Schaefer, Claudia Lengerke

**Affiliations:** 1 University of Basel and University Hospital Basel, Department of Biomedicine, Basel, Switzerland; 2 University Hospital Basel, Division of Hematology, Basel, Switzerland

**Keywords:** Stem cells, Post-translational modifications

## Abstract

Research of the past view years expanded our understanding of the various physiological functions the cell-fate determining transcription factor SOX2 exerts in ontogenesis, reprogramming, and cancer. However, while scientific reports featuring novel and exciting aspects of SOX2-driven biology are published in near weekly routine, investigations in the underlying protein-biochemical processes that transiently tailor SOX2 activity to situational demand are underrepresented and have not yet been comprehensively summarized. Largely unrecognizable to modern array or sequencing-based technology, various protein secondary modifications and concomitant function modulations have been reported for SOX2. The chemical modifications imposed onto SOX2 are inherently heterogeneous, comprising singular or clustered events of phosphorylation, methylation, acetylation, ubiquitination, SUMOylation, PARPylation, and O-glycosylation that reciprocally affect each other and critically impact SOX2 functionality, often in a tissue and species-specific manner. One recurring regulatory principle though is the canonical PI3K/AKT signaling axis to which SOX2 relates in various entangled, albeit not exclusive ways. Here we provide a comprehensive review of the current knowledge on SOX2 protein modifications, their proposed relationship to the PI3K/AKT pathway, and regulatory influence on SOX2 with regards to stemness, reprogramming, and cancer.

## An introduction to SOX2 and underexplored aspects of its molecular regulation

The SOX/Sox (SRY homology box) family of proteins comprises 20 individual members in man and mouse [1], of which SOX2/Sox2 is the most explored. SOX proteins are principally defined by a conserved DNA-binding element, the so-called high mobility group (HMG) that relates to a transcriptional master regulator of virility (i.e., *SEX determining factor Y*, SRY) and thus functionally qualifies SOX/Sox proteins as DNA-binders [2]. Accounting for HMG-residue conservation, the SOX family branches into eight sub-categories (SoxA-H), of which SoxB further divides into SoxB1 and SoxB2 groups reflecting more recent gene duplication events [3, 4]. While Sox proteins contribute to various cellular functionalities, reprogramming capacity is largely confined to members of the SoxB1 group (i.e., Sox1, Sox2, and Sox3) [5]. Early reporter assays involving designed plasmid-borne promoter-reporter constructs assigned an expression co-modulatory activity to C-proximal parts of SOX2, termed “trans-activating domain” (TAD) [6]. However, while the HMG is an evolutionary conserved folding unit defined by cognate sequence elements and structurally resolved in considerable molecular detail (see PDB deposits 1O4X and 2LE4), the TAD remains a functional concept not yet supported by structural data.

Noteworthy, SOX2 imposes transcription modulatory significance often in conjunction with co-factors [7] such as OCT3/4 in ES cells [8, 9] or PAX6 in the visual system primordia [10]. Accordingly, while the well-resolved N-terminus with its contained HMG-domain and superimposed nucleo-cytoplasmatic transport motifs (nuclear localization sequence (NLS) [11] and nuclear export sequence (NES) [12], respectively) provides for proper subcellular distribution and DNA-binding, the C-terminus is assumed to engage in interactions with co-factors. While this concept is compelling, the SOX2 C-terminus nevertheless lacks secondary structural elements classically associated with protein–protein interactions such as ANK-repeats, SH2-, WW-, Bromo-, or RING-domains (to name but a view). Indeed, despite upward 6900 peer-reviewed scientific contributions featuring various aspects of SOX2 biology (pubmed, status June 2019), the protein’s C-terminus remained structurally enigmatic and eludes classical sequence motif prediction tools.

By contrast, the HMG/DNA-interaction has been resolved in considerable detail and a DNA recognition consensus long been defined for SOX2/Sox2 (i.e., CCCATTGTTC in man and CTTTGTC in mouse) [13, 14]. However, with the central TTGT element being the preferred recognition motif of all SOX/Sox proteins and several thousand copies of the extended consensus in the human genome, SOX2 resembles a rather promiscuous transcription factor. A certain degree of selectivity however is imposed by co-factors (see above), although many of which are likewise shared amongst Sox proteins [15, 16]. In reality therefore, the current loading status of an individual consensus site in a particular *SOX2*^*+*^ cell remains effectively unpredictable. To give an example: While an in silico search of the glioblastoma cancer genome identified 4883 potential SOX2 docking sites in advanced human glioma, altered RNA expression was subsequently confirmed for no more but 489 protein coding genes and 105 pre-miRNAs by Chip-seq [17]. Underscoring these numbers, 699 significantly modulated SOX2 downstream targets were more recently identified in nasopharyngeal cancer cells by cDNA microarray [18]. Nevertheless, also the mere occupation of a binding site without concomitant expression modulation of juxtapositioned genes may be a regulatory function, as secondary effects amongst downstream targets may be suppressed this way. Vice versa, expression changes faithfully assigned to SOX2 must not inevitably relate to its presence at a given locus, but may reflect indirect effects. In any case though, SOX2-driven phenotypes, often laid out to the reader as linear causal narratives, are almost inevitably multifactorial.

Interestingly, also the organization and regulation of the *SOX2* gene on chromosome locus 3q26.3-q27 are remarkable and in their functional consequences not fully resolved yet. Not only can SOX2 potentiate the expression of its own gene as part of autoregulatory feed-forward mechanisms [19], the single exon *SOX2* moreover falls into the intron of a much greater, superimposed genetic element called *SOX2OT* (SOX2 overlapping transcript). This peculiar gene arrangement is phylogenetically conserved between man and mouse, where *Sox2ot* was recently shown to modulate Sox2 expression in cortical neuronal progenitors, indeed [20]. This finding may be of particular relevance, as near every analysis of *SOX2/Sox2* involves knock-down or knock-out technology, whereas potential side-effects on *SOX2OT* are rarely considered. Vice versa, an experimental outcome faithfully assigned to *SOX2* may further involve *SOX2OT* functional contributions.

## Functional roles of SOX2

SOX2 is probably best known for its technical application in reprogramming where, in conjunction with co-factors (e.g., OCT4, KLF4, and cMYC [21, 22] or subsets thereof [5, 23]), SOX2 enables the derivation of human or murine induced pluripotent stem cells (iPSCs) from terminally differentiated somatic cells. Although a milestone in present day cell biology and clearly of unprecedented potential for future biomedical application, the enforced overexpression of a subset of exogenously introduced transcription factors in otherwise terminally differentiated body cells remains an artificial intervention, and (more relevant in the given context) remains mechanistically not fully elucidated with regard to specific contributions of SOX2.

More instructive in this sense are murine in vivo analyses that illustrate a pivotal physiological significance of Sox2 throughout the vertebrate life cycle, from early embryogenesis to adult tissue homeostasis [24]. In particular: Deletion of Sox2 in the zygote results in early embryonic lethality with concomitant malformation of the epiblast [25]. Throughout morulation, SOX2 expression coincides with pluripotent embryonic stem (ES) cells that later localize to the inner cell mass of the blastula. Underscoring a critical role in ES cell maintenance, both deletion and overexpression of Sox2 impose ES cell differentiation in vitro [8, 26]. In the developing embryo, Sox2 expression is maintained in lineage committed, multipotent progenitor cells mostly of neuronal or epithelial fate [24]. These ontological paths are probably best resolved for the developing (central) nervous system. Here, Sox2 expression has been traced from the cerebral tubing in early embryogenesis to the ventricular layer of the neuronal cortex (where neural stem/progenitor cells reside at mid-fetal development) and finally to the subventricular zone of the lateral ventricle and the granular zone lining the hippocampus (where Sox2 positive NSCs locate in the adult brain) [27]. Another well-defined ontological branch concerns the developing gastro-intestinal tract, where Sox2 expression associates with the engulfing foregut and derived endodermal structures from which esophagus and anterior stomach evolve [28]. In the developing skin, Sox2 positive cells are first detected in the mesenchymal condensates giving rise to the dermal sheath and the dermal papilla [29], while at adulthood Sox2 marks multipotent skin stem cells with multilineage differentiation capacity [30, 31]. Sox2 is further instrumental for proper dental development [32], while its expression in murine optic cup [33] and retina [34] progenitors proved critical for eye development. Underscoring conserved functions in man, hereditary *SOX2* haplo-insufficiency was associated with syndromic microphthalmia type 3 (MCOPS3) [35], a disease characterized by micro- or an-ophthalmia and concomitant cognitive and/or developmental impairment [36]. Noteworthy, a strong dependence on Sox2 expression also persists in adulthood as illustrated by an inducible murine *Sox2* knock-out model [37] that shows enforced tissue cell apoptosis and onset of lethality within two weeks of Sox2 depletion.

During the last decade, aberrant SOX2 expression has been further associated with various forms of cancer, particularly of epithelial or neuronal origin [38–46], for review see [47, 48]. In some but not all of these SOX2 has been linked to cancer stemness. In patients with breast or ovarian cancer for example, SOX2 induction is already observed at early disease stages and further associates with disease progression, metastasis, and relapse [40, 43, 45, 46]. In glioblastoma as another example, elevated SOX2 expression associates with increased cell motility and tumor spreading and is also detected amongst circulating CSC islets [49–51]. Further involving SOX2 in cancer stemness, elevated SOX2 expression associates with chemotherapy-resistance effects [52], induces stemness and endothelial-to-mesenchymal-transition gene signatures [17], and promotes clonogenicity and in vivo tumorigenicity in respective model systems [43, 52]. In sum, various lines of experimental evidence underscore connectivity between SOX2 expression and CSCs in some cancers, although likely not all SOX2-positive cancer cells are CSCs and vice versa. Overall the CSC concept is controversially discussed in solid tumors, and plasticity has been noted between stem and non-stem cells [53].

Depending on disease entity and state, aberrant *SOX2* expression may either result from gene duplication events or dysregulated transcription of the endogenous locus [47, 48]. As a consequence, divergent focally restricted to ubiquitous intra-tumoral distribution of SOX2 expression has been reported. In cancers in which SOX2 expression associates with stemness, SOX2 occurs mostly in the absence of gene amplification. By contrast, in squamous cell lung carcinoma, SOX2 expression frequently coincides with amplifications at its chromosomal locus, so that SOX2 stains rather homogenously in tumor biopsies and has been described as a lineage-survival factor [38, 54]. Here, SOX2 obviously cannot be considered a distinguishing marker of the CSC compartment.

What is more, although near univocally described as an oncogene and driver of transformation, in gastric and non-small lung cell carcinoma SOX2 expression was in fact reported as a favorable molecular prognostic factor [55–59]. This remains tissue specific and might relate to even more aggressive phenotypes mediated by alternative pathways or external stimuli. As such, *Helicobacter pylori* infections was shown to influence SOX2 expression in gastric cancer [60, 61], where SOX2 has been described as a trans-activator of PTEN, thus inhibiting PI3K/AKT-driven cell cycle progression and anti-apoptosis effects [55, 62, 63]. External influences also concern non-small cell lung cancer, where a smoking-induced injury response [64] was shown to trigger a premalignant state characterized by PI3K induction and molecularly uncoupled SOX2 expression in basal epithelial cells [64], while in dysplasia expression of SOX2 may depend on FGF triggered MAPK/ERK signaling [65].

**Fig. 1:**
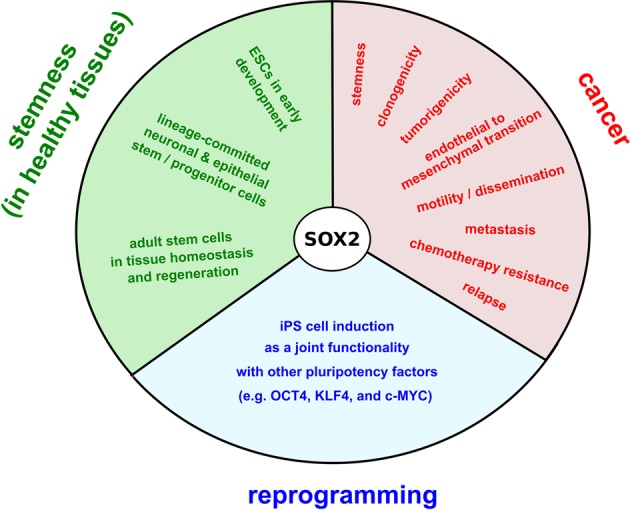
The versatile roles of pluripotency factor SOX2 in stemness, reprogramming, and cancer. The transcriptional master regulator SOX2 *(SEX determining region (SRY) homology box 2)* relates to various aspects of naturally occurring, healthy stem and progenitor cells (green), pluripotency induction amongst terminally defined somatic cells (blue), and cancer stemness and progression (red). With regards to stemness in healthy tissues, the protein sustains pluripotency amongst embryonic stem cells (ESCs) in early embryogenesis, co-segregates with lineage committed stem/progenitor cells of mostly epithelial or neuronal fate in advanced ontogenesis, and finally co-localizes with tissue-resident stem cells that warrant for tissue homeostasis and regeneration in adulthood (green). Moreover, SOX2 is an indispensable reprogramming factor that in conjunction with co-factors drives the conversion of terminally differentiated somatic cells into induced pluripotent stem cell conditions (iPSCs, blue). Finally, SOX2 is a powerful oncogene that functionally relates to cancer stemness and various further hallmark functionalities amongst which clonogenicity, tumorigenicity, endothelial-to-mesenchymal transition (EMT), cancer cell mobility, tumor cell dissemination, metastasis, chemotherapy resistance, and relapse (red)

Taken together, SOX2 is a cell-fate determining transcription factor that has been functionally implicated in the induction and maintenance of pluripotent iPS and ES cells, multipotent lineage-committed progenitors, and tissue stem cells of mostly epithelial or neural fate. Moreover, SOX2 has been recognized as a powerful oncogene in various cancer types, where it regulates CSCs and functionally relates to several other hallmarks (see Fig. 1 for schematic overview of SOX2 functionality). While the cell biological relevance of SOX2 thus appears clearly defined, the molecular understanding of SOX2 protein regulation remains surprisingly fragmental and comprises various aspects of effective uncertainty, as can be deduced from the examples given above.

## The PI3K/AKT signaling pathway

Key cell biological features such as growth, proliferation, and survival are functional manifestations of highly intertwined underlying molecular networks probably best illustrated by systems biology approaches [66]. Although a crude underestimation of cellular complexity, such cell-biological features have been historically assigned to a hand-full of major cognate signaling cascades or pathways, amongst which the PI3K/AKT axis [67]. This molecular module converts extracellular stimuli (mostly receptor/ligand interaction or ligand-imposed receptor dimerization events at the plasma membrane) via a phosphorylation cascade into molecular downstream responses, and furthermore relates to upward mTORC2 and downstream mTORC1 complexes as associated regulatory relays. Although a myriad of cell-type specific input and output functions have been assigned to the pathway [67], an evolutionary conserved primordial functionality of the PI3K/AKT axis is that of a nutrient sensor. Triggered by insulin or amino acid availability on the cell surface, the PI3K/AKT axis converts information on the current bioavailability of these metabolites into downstream effector functions within the cell [68–72]. In response to insulin for example, PI3K/AKT modulates cellular glucose uptake (more actually the cell surface expression of individual glucose transporters, GLUT) involving AS160, DGKzeta, and PIP5K proteins [73, 74]. In response to amino acids such as glutamate, PI3K/AKT stimulates mTORC1, the mammalian target of rapamycin complex 1, that induces phospho-activation of ribosomal protein S6 (RPS6) by S6 kinase (S6K1) [75, 76] and a release of eIF4E from its inhibitor 4E-BP1 [77, 78] to launch protein synthesis. Further prominent effector functions to which PI3K/AKT signaling relates, either in direct or indirect terms, comprise cell cycle progression, (anti)-apoptosis, and more [67]. Accordingly, mechanisms that overactivate the PI3K/AKT axis or impair its inactivation (such as mutations in *PIK3CA* and/or *PTEN* genes, for example) range amongst the most prominent molecular aberrations in cancer and resemble mutational hot spots for resistance development [79, 80].

Interestingly, cancers of immanent high SOX2 expression often show intrinsically high mutations rates in *PI3KCA* or *PTEN* genes as well [50, 64], thus suggesting functional connectivity between PI3K/AKT signaling and stemness-inductory roles of SOX2.

## Intertwined relationships between PI3K/AKT signaling and SOX2 in stemness

Cell growth and cell proliferation are two phenotypically entangled but mechanistically rather distinct processes. While growth largely depends on PI3K/AKT/mTORC1-imposed protein synthesis (see above), proliferation is a principle reflection of cell-cycle progression and thus cyclin kinase activities [81, 82]. Although these processes are easily experimentally uncoupled in vitro (e.g., with the mTORC1 selective inhibitor rapamycin or else with cyclin kinase inhibitors), such distinction is much harder to make in vivo. Classical examples however comprise arborization (i.e., the outgrowth of dendritic and axonal connections amongst central nervous cells in early postnatal development) [83], which represents an extreme case of cell mass enrichment (growth) at near persistent cellularity. By contrast, the initial stages of embryogenesis comprise a rapid succession of cell division events that occur essentially without size increase (i.e., the diameter of oocyte and blastula is near invariant) [84]. Accordingly, in cells that constitute the morula or the inner cell mass of the blastula (i.e., ESCs), protein synthesis evidently is attenuated to a degree that neither supports mass enrichment (growth) nor differentiation. These adaptations appear conserved in resting tissue stem cells, where tightly constrained protein synthesis is likewise required to suppress differentiation and thus sustain stemness. In line with these notions, somatic stem cells are generally smaller (with size being an indicator of protein synthesis) and show lower protein formation rates than surrounding tissue cells [85, 86]. Noteworthy, while the PI3K/AKT/mTORC1 axis clearly relates to protein formation, also SOX2 has been implicated in these processes, as a SOX2-imposed translation factor, eIF2A, initiates protein synthesis from unconventional start sites within the normally untranslated 5′-UTR of proto-oncogenes [87].

As growth and differentiation become inevitable, e.g., in the developing embryo or injury settings, protein synthesis must be enforced amongst stem cells or their derived progeny. Consistently, PI3K activity has been further associated also with cellular differentiation (e.g., the conversion between luminal and basal layers of the mammary epithelium) [88]. In cancer finally, such growth regulatory mechanisms may be largely uncoupled and overruled by mutations inducing and sustaining clonal expansion (e.g., involving aberrant PI3K/AKT/mTORC1 signaling). The PI3K/AKT pathway has thus been long recognized as potential target for therapeutic intervention in cancer [80, 89], and very recently, a combination of the PI3K-alpha selective inhibitor BYL719 (alpelisib, Pigray®) with the estrogen receptor antagonist Fulvestrant® was approved for the treatment of advanced mammary carcinoma, as this treatment nearly doubled the median progression-free survival in *PIK3CA*-positive *HR*^*+*^*/HER2*^*+*^ breast cancer patients (SOLAR-1 trial, FDA approval: May 24th, 2019).

While these recent developments underscore the therapeutic potential of PI3K/AKT inhibition in general, the particular significance of *SOX2*^*+*^ CSCs as a cellular reservoir of disease relapse has not been therapeutically exploited thus far. This is remarkable, as several lines of experimental evidence gathered in healthy and transformed cell matter, clearly underscore an interdependence between PI3K/AKT signaling on the one hand and SOX2-imposed (cancer) stem cell characteristics on the other: Mutations in *PTEN* for example support the outgrowth of healthy and malignant mammary epithelium derived stem cells, which can be antagonized by the PI3K/AKT cross-reactive inhibitor perifosine [90]. Effectively separating these effects, either inhibition of PI3 kinase or knock-down of downstream AKT1 were later shown to impair the survival of tumor initiating supposed breast carcinoma stem cells [91].

An immediate molecular connectivity between Akt and Sox2 however was first established in mouse where Akt-imposed phosphorylation supports Sox2 driven transcription amongst ESCs [92] and functionally cooperates with Oct4, Klf4, and c-Myc in the induction of iPSCs [93]. These observations were further substantialized in man, where AKT-driven phosphorylation was shown to sustain SOX2 protein stability, nuclear localization, and thereby regulate in vitro clonogenicity and in vivo tumorigenicity of breast carcinoma cells [52]. Similar results were reported for nasopharyngeal cancer cells, albeit with a less well defined AKT-inhibitory reagent, DC120, that blocks AKT in its Thr308 and Ser473 phosphorylated state [94]. Further validating AKT as a conserved upstream regulator of SOX2 expression, AKT pathway inhibition or siRNA mediated knock-down reduced SOX2 levels also in esophageal squamous cells [95, 96]. While one report proposes nitrosylation of AKT as a regulatory principle in SOX2 expression [95], the other describes ubiquitin-ligase UBR5 as a mediator of SOX2 protein turnover in absence of AKT kinase activity [96].

The dependencies between PI3K/AKT and SOX2 however are reciprocal, as probably best exemplified in glioblastoma, where PI3K/AKT-imposed phosphorylation sustains nuclear SOX2 expression as a driving force of tumor cell dissemination, while in turn SOX2 supports *PI3KCA* gene expression (which actually locates in near juxtaposition of the *SOX2* locus on chromosome band 3q26-28) and subsequent downstream activation of AKT [50]. Similarly, SOX2 and KLF4 jointly drive *PIK3CA* expression in nasopharyngeal carcinoma, thus enhancing PI3K/AKT activity and tumorigenesis, while overexpression of *PI3KCA* rescues growth inhibitory effects imposed by *SOX2* knock-down [18]. In Ewing’s sarcoma finally, the disease characteristic EWS1/FLT1 gene fusion has been identified as an inductor of *SOX2* expression, which functionally correlates with enforced cell proliferation, anti-apoptosis, and enhanced PI3K/AKT signaling [97].

Interestingly, an interconnectivity between SOX2 and PI3K/AKT is also evident in reprogramming settings, despite the fact that iPS cell induction involves artificial overexpression of exogenously introduced SOX2/Sox2. While enforced SOX2 expression coincides with elevated AKT activity during reprogramming [98], PI3K/AKT signaling provides critical contributions to iPS cell survival also thereafter [99]. The endogenous *Sox2* locus however seems to be the target of regulatory feedback inhibition, as in reprogramming settings AKT1 displaces gene activatory FoxO1 from *Sox2*, whereas FoxO1 is re-activated and endogenous *Sox2* expression restored upon inhibition of AKT [98].

**Fig. 2:**
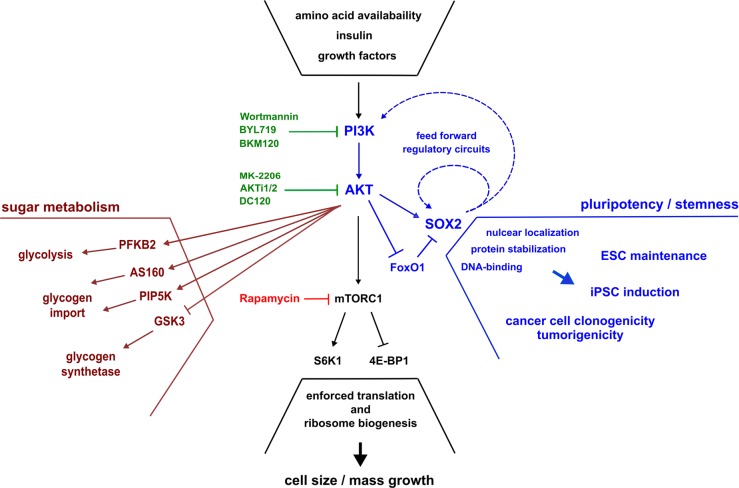
The PI3K/AKT/SOX2 signaling axis is a conserved module in stemness, reprogramming, and cancer. The PI3K/AKT pathway converges extracellular stimuli conferred via respective receptors on the plasma membrane into downstream effector functions within the cell. Classical PI3K/AKT mediated phenotypes involve adapted sugar metabolism in response to insulin receptor stimulation (maroon) and aggravated protein synthesis in response to amino acid availability, as sensed by e.g., the glutamate receptor and mediated by the mTORC1 complex (black). Noteworthy, both PI3 and AKT kinases relate to various further cell biological features amongst which cell cycle control, (anti)-apoptosis, DNA damage response, nitric oxide signaling, and others (not depicted), while yet another functionality characteristic of SOX2 positive cells has been identified in the PI3K/AKT/SOX2 axis (blue). This signaling branch critically determines SOX2 protein stability, nuclear localization, and DNA regulation, and thus functionally relates to sustained pluripotency amongst naturally occurring embryonic stem cells (ESCs), the conversion of terminally differentiated body cells into induced pluripotent stem cells (iPSCs), and furthermore critically contributes to cancer initiation, progression, and relapse. SOX2 has been identified as a direct target of AKT-imposed phosphorylation, and a functional dependence on PI3K/AKT signaling experimentally validated involving various inhibitory reagents (green) directed against either AKT or further upstream PI3K. By contrast, inhibition of downstream mTORC1 (rapamycin, red) does not immediately affect SOX2 protein characteristics. However, the PIK3/AKT pathway involves various feed-back and feed-forward regulatory loops, amongst which SOX2 imposed transcriptional auto-stimulation and a reciprocal induction of *PI3KCA* gene expression (dotted blue lines)

These various interdependencies between PI3K/AKT and SOX2, detected across a spectrum of different malignant and healthy (stem) cell types, have led to the notion of an independent PI3K/AKT/SOX2 signaling branch that diverges from the canonical PI3K/AKT/mTORC1 axis (see refs. [100, 101] and Fig. 2 for schematic illustration). This distinction is underscored by experimental evidence indicating that various AKT or PI3K inhibitors, but not the mTORC1 selective inhibitor rapamycin, affect SOX2 protein homeostasis in several tumor entities [50, 52]. However, inhibition of mTORC1 has also been described to attenuate SOX2 and further downstream SOX9 expression in glioma [102], and both PI3K inhibitors and rapamycin reported to suppress SOX2-driven tumorigenicity from esophageal squamous cells in a murine xenografts [103]. These discrepancies might be explained by the fact that the PI3K/AKT signaling pathway involves various feed-forward and feed-backward mechanisms, so that rapamycin may indeed regulate SOX2, albeit in indirect terms.

Putting these observations in an even greater context, a mass spectrometry based systematic phosphorylation site analysis identified several AKT-imposed modifications not only in SOX2, but also in other pluripotency inducing transcription factors [104]. Intriguingly, many of these sites superimpose with DNA-binding elements or sequence stretches functionally linked to nucleo-cytoplasmatic transport (NLS, NES), thus suggesting AKT-imposed phosphor-modification as a shared regulatory principle of subcellular distribution and chromatin binding amongst pluripotency inducing TFs [104, 105]. Further supporting these notions, a glycoproteomic analysis performed to identify surface molecules associating with pluripotency, retrieved *leucine-rich repeat neuronal progenitor 1 (LRRN1)* as a factor critically regulating SOX2, OCT4, and NANOG protein localization and turnover in hESCs [106]. Intriguingly, shRNA-mediated knock-down of *LRRN1* associated with impaired AKT activity and thereby nuclear depletion and proteasomal degradation of SOX2, OCT4, and NANOG [106]. While these findings underscore AKT-imposed phosphorylation as a common regulatory determinant of pluripotency TFs, they moreover indicate that AKT-dependent regulation of SOX2 protein turnover is a conserved functional feature shared also with healthy (ES) cells.

While the molecular regulation of most other SOX proteins remains poorly defined, further connectivity to the PI3K/AKT axis has been proposed for SOX4. In mammary carcinoma, elevated SOX4 expression promotes PI3K/AKT signaling [107], while glioblastoma cell cycle progression involves SOX4-dependent modulation of AKT and p53 [108]. Interestingly, SOX4 and SOX2 proteins only distantly relate within the SOX family [3], but the two proteins show nearly overlapping occurrence in human cancers [48]. In mammary carcinoma and glioblastoma stem cells specifically SOX4 has been implicated as a regulator of SOX2 [109, 110], suggesting that the observed connectivity of this factor with PI3K/AKT signaling may also reflect an indirect regulatory circuitry further involving SOX2.

Taken together, various lines of evidence elaborated in mouse models, human cancer cells, reprogramming settings, and clinical datasets, exemplify a functional cooperativity of SOX2 with the PI3K/AKT signaling pathway. Although divergent in detail, two recurrent features are noted: (i) nuclear localization and protein stabilization of SOX2 depend on AKT kinase activity, and (ii) transcription of *PIK3CA*, the gene encoding the catalytic subunit of PI3Kα, is re-enforced by SOX2.

## SOX2 secondary modifications

**Fig. 3:**
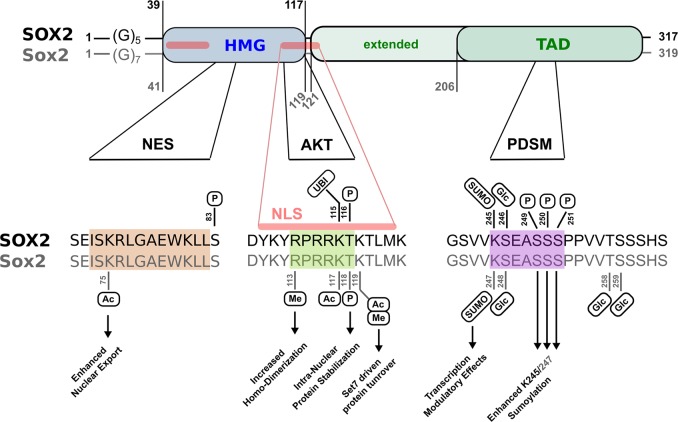
SOX2/Sox2 is a molecular target of various secondary modifications. Human (top) and murine (bottom) SOX2/Sox2 protein sequences contain a N-terminal DNA-binding domain (high mobility group, HMG, blue), a C-terminal trans-activation domain (TAD, green), and (due to a slightly deviant glycine repeat near the proteins’ start) differ in length by two amino acids. Note that the TAD is a functional concept, not a sequence- or structure-based domain signature, so that segments of variable length have been annotated as such, with longest forms commencing right after the HMG. Conserved functional elements are indicated: nuclear export signal (NES), nuclear localization signal (NLS, red), AKT-recognition consensus (AKT), and phosphorylation-dependent SUMOylation motif (PDSM). Note that the NLS (red) comprises two separate stretches, of which the latter superimposes with the AKT consensus motif near the C-terminal end of the HMG domain. NES, NLS/AKT, and PDMS sequences are further detailed below with indication of the particular chemical modifications identified thereat: phosphorylation (P); acetylation (Ac); methylation (Me); ubiquitination (UBI); SUMOylation (SUMO); O-GlcNAcylation (Glc). Wherever known, corresponding functional manifestations are indicated (arrows). Note that further putative acylation sites (i.e., residues K37, K60, K67, K89, K97, K105, K111, and K123) have been proposed for Sox2, but not functionally validated thus far. Moreover, SOX2 is a target of PARPylation, while the specific site of this modification remains to be determined

Throughout the past years, various examples of SOX2/Sox2 protein modifications have been identified, but not comprehensively summarized in standard protein data depositories such as UniProt/Swiss-Prot/ExPASy (status June 2019). Protein biochemical modifications reported for SOX2/Sox2 comprise phosphorylation, acetylation, methylation, ubiquitination, SUMOylation, PARPylation, and O-GlcNAcylation events that are successively described in the subsequent sections, systematically listed in Table 1, and their relative localization and functional implications schematically illustrated in Fig. 3.

**Table 1:** Compendium of the secondary modifications imposed onto SOX2/Sox2

Type of modification	Species	Site	Study matter	Functional significance	References
**Phosphorylation**	Man/Mouse	THR 116 Thr 118	Murine ESCs/iPSCs various human cancer- derived cell types	AKT-dependent intra-nuclear SOX2 protein stabilization, as a functional prerequisite of ESC maintenance & cancer cell clonogenicity/tumorigenicity	Jeong et al. [93] Fang et al. [92] Schaefer et al. [52] Qin et al. [94]
	Man	SER 83	Heterologous expr. in Sf9 insect cells	Uncertain	Malak et al. [104]
	Man	SER 249 SER 250 SER 251	Human ESCs	Phosphorylation-dependent SUMOylation (PDSM) of nearby LYS 245	Van Hoof et al. [111] Rigbolt et al. [112]
**SUMOylation**	Man/Mouse	LYS 245 Lys 247	Human ESCs Murine ESCs	Modulation of target gene expression	Van Hoof et al. [111] Tsuruzoe et al. [113]
**Acetylation**	Mouse	Lys 75	J1 mouse ESCs	Modulation of NES functionality and protein decay	Baltus et al. [12]
		Lys 37, 60, 67, 75, 89, 97, 105, 111, 117, 119, 123		Unknown - Lys 117ac may compete with LYS 115ub - Lys 119ac may compete with Lys 119met	
**Methylation**	Mouse	Arg 113	Murine embryonic carcinoma P19 cells	Regulation of homo-dimerization and chromatin binding	Zhao et al. [117]
	Mouse	Lys 119	Murine ESCs	Set7-imposed methylation fosters WWP2-driven ubiquitination and decay	Fang et al. [92]
**Ubiquitination**	Man	LYS 115	Esophageal squamous cancer cell lines	UBR5-imposed ubiquitination & protein turnover	Wang et al. [96]
**PARPylation**	Mouse	unknown	Murine ESCs	Modulation of target gene expression in ES cell differentiation	Gao et al. [118]
**O-GlcNAcylation**	Man/Mouse	SER 246 Ser 248	Murine ESCs/iPSCs S2VP10 human pancreatic cancer cells	Implicated in ESC maintenance and tumorigenesis	Jang et al. [123] Myers et al. [119] Sharma et al. [124, deposited]
	Mouse	Thr 258 Ser 259	E14 murine ESCs	Uncertain	Jang et al. [123]

Systematic tabular compilation of the various protein secondary modifications hitherto described for pluripotency factor SOX2/Sox2 with indication of modification site (human residue no., capitalized; murine reside no., regular), the cell type the particular modification was identified in (ESC, iPSC, cell lines), its proposed functional significance, and respective reference articles

## SOX2 protein phosphorylation

Of all secondary modification assigned to SOX2, phosphorylation is by far best documented. This modification primarily concerns residue Thr116 (alias Thr118 in mouse; note that the murine protein is two residues longer than its human orthologue) that is part of an AKT recognition motif (RPRRX-**S/T**). This sequence stretch superimposes with the nuclear-localization sequence (NLS) near the C-terminal end of the HMG-domain [11], suggesting that phosphorylation of Thr116 may modulate SOX2 subcellular distribution. Interestingly, the RPRRX-T signature is stringently conserved only amongst SoxB1 proteins (i.e., Sox2 and its closest paralogues Sox1 and Sox3) [3]. In other Sox proteins by contrast the motif is either functionally compromised by loss of a hydroxy acceptor chain (SoxF), structurally distorted by insertion of proline residues (SoxB2, SoxG, and SoxH classes), charge modified by arginine to glutamine replacement (SoxE), or further derivatized by exchange of the acceptor threonine for serine (Sox12 and Sox15) thus modifying the steric context of the phosphorylation site.

Underscoring the concept of AKT-imposed SOX2 modification, a physical interaction between AKT/Akt and SOX2/Sox2 proteins was independently demonstrated in murine and human cells [52, 93]. However, while phosphorylation of mouse Thr118 sustains Sox2 driven transcription in ES and iPS cells [93], alanine replacement analysis revealed that Thr116 is not stringently required for the nuclear localization of human SOX2 [52]. What is more, also a mass spectrometry based systematic phosphorylation site analysis involving heterologous expression of SOX2 and AKT in Sf9 cells, did not detect AKT imposed phosphorylation at residue Thr116 [104]. Instead, it uncovered a further, hitherto unrecognized AKT-imposed phosphorylation at Ser83, the functional relevance of which however remains to be determined.

In sum, AKT activity unquestionably sustains SOX2 nuclear localization, DNA binding, and prevents SOX2 from proteasomal decay in the cytosol [52, 96]. However, while a phosphorylation of the indicated residues has been repeatedly linked to AKT (see above), it remains a matter of debate, if AKT imposed phosphorylation indeed regulates SOX2 nuclear import (i.e., the association with a nuclear import adapter or transporter) and if this functionality alone has a decisive significance in these regards. Noteworthy, various further modifications have been associated with the nucleo-cytoplasmatic shuttling of SOX2, and residue Thr116 furthermore linked to SOX2 protein turnover.

## Phosphorylation-induced SUMOylation of SOX2

Additional phosphorylation sites described for SOX2 comprise the residues Ser249, Ser250, and Ser251 originally identified in a phospho-proteomic analysis of human ESCs [111]. Of these, Ser251 was reconfirmed in a subsequent independent phospho-proteomic analysis in the same cell type [112]. Proposed as being functionally redundant [111], all three phosphorylation sites relate to nearby Lys245/247 that is a target of protein SUMOylation [113]. While a conservative amino acid replacement at this position (i.e., K247R) has no evident effect on Sox2 protein stability or localization, SUMOylation at Lys247 coincides with reduced Sox2 binding to an Fgf4 enhancer element in the context of Oct3/4 co-expression, so that a transcription modulatory significantly was proposed for this residue modification [113].

Documenting functional connectivity of phosphorylation and nearby SUMOylation events in this segment, a SOX2(S249-251D) mutant mimicking constitutive phosphorylation at the indicated residues strongly induced SUMOylation of SOX2, whereas a phosphorylation incompetent SOX2(S249-251A) mutant impairs this modification. SUMOylation was fully obstructed though only in a SOX2(K245A) mutant, thus reconfirming Lys245 as the target site for SOX2 SUMOylation [111]. These data jointly define a phosphorylation-dependent SUMOylation motif (PDSM, ΨKxExxSP) in SOX2, as previously described also for SOX3, SOX8, and SOX9 [114], albeit in slightly different forms (i.e., SOX2 contains an additional amino acid insertion between SUMOylation and phosphorylation sites, so that the motif was initially overseen).

Noteworthy, although AKT is a stereotype Ser/Thr kinase and the PI3K/AKT pathway indeed activated in ES cells [111], a functional involvement of AKT in PDSM phosphorylation appears unlikely as no corresponding signature peptides were identified in an AKT/SOX2 co-induction MS analysis [104] and none of the above described mutants shown to impair nuclear localization [111, 113]. While a cognate kinase thus remains to be defined, the phosphorylation status of Ser249, Ser250, and Ser251 did not significantly change upon addition of differentiation-inducing BMP (bone morphogenic protein) to hESCs, suggesting that the PDSM mechanism—although a modulator of transcription [113]—may be overruled by even more dominant regulatory elements [111].

## Acetylation of the SOX2 NES motif

As p300/CBP-imposed protein acetylation and HDAC3-related deacetylation had been implicated in the nucleo-cytoplasmatic distribution of SRY [115], also the Sox2 protein was scanned for acetylation events in a two-armed MS-based approach [12]. This analysis uncovered p300/CBP-imposed in vitro acetylation on various Sox2 sites including lysine residues K37, K60, K67, K75, K89, K97, K105, K111, K117, K119, and K123 that all match the HMG domain except for K123. While not all of these modifications may occur in vivo—even more unlikely in stringent coupling—residue K75 was noted to fall into the nuclear export sequence (NES) of Sox2, and the functional relevance of this site thus further examined. The Sox2 NES motif was identified on basis of sequence homology to Sry and Sox9 [12], where a nuclear export activity had first been identified and functionally validated by impaired sexual differentiation in mice expressing NES-mutant alleles [116]. Interestingly, just as the NLS motif, also the NES sequence locates to the HMG domain and is phylogenetically conserved between SRY and SOX/Sox proteins [12]. Confirming functional relevance, an interaction with the nuclear export receptor, Crm1, is impaired in NES mutant forms of Sox2 thus causing their subcellular mislocalization [12]. Finally connecting these findings to acetylation events, K75A (acetylation null) and K75Q (acetylation mimicry) alleles were cloned and found to modulate Crm1-docking and Sox2 nucleo-cytoplasmatic distribution, indeed [12]. Moreover, deacetylating reagents retain Sox2 in the nucleus and sustain target gene expression, whereas elevated acetylation enforces nuclear export resulting in increased Sox2 protein decay [12].

## Methylation and methylation-imposed ubiquitination of SOX2

A physical association of Sox2 with *co-activator associated arginine methyltransferase 1 (CARM1)* has been reported for murine embryonal carcinoma P19 cells, and CARM1-imposed Sox2 methylation at residue Arg113 further investigated in breast carcinoma MCF7 cells [117]. A methylation at this site was proposed to enforce Sox2 homo-dimerization and functionally linked to the expression of Sox2 target genes, while chromatin binding blocks methylation of arginine 113 [117].

Moreover, a phosphorylation/methylation switch has been proposed to modulate Sox2 protein stability in murine ESC maintenance and differentiation, and two E3-ligases, WWP2 and UBR5 implicated in the ubiquitination-dependent decay of Sox2/SOX2, albeit in a species- and tissue-selective manner [92, 96]. Jointly, these reports confirm AKT/Akt as a driving force of SOX2/Sox2 nuclear localization and cell-fate determination, and cytosolic proteasomal degradation of SOX2/Sox2 in absence of AKT/Akt activity. While these notions were first elaborated in murine ESCs [93] and human cancer cells (e.g., breast and esophageal carcinoma cell lines) [52, 94], Wong and co-workers embed these findings into following intriguing regulatory concept: The aforementioned Thr116/118 phosphorylation site is flanked by two lysine residues (one on either side, see Fig. 3 for schematic illustration). The C-proximal of these residues (Lys119) is subject to Set7 imposed protein methylation that fosters WWP2-driven ubiquitination and cytosolic proteasomal decay of Sox2. These processes emerge during ESC differentiation, but compete with stemness sustaining Akt1-driven phosphorylation of neighboring Thr118 [92]. While the exact ubiquitination site was initially not resolved, it may have been identified in a more recent publication by Wong and co-workers in human esophageal cancer cells [96]. Here, the authors describe a competition between AKT1 imposed phosphorylation on Thr116 (the human paralogue of murine Thr118) and UBR5-driven ubiquitination of its N-proximal Lys115. While both lines of evidence have been elaborated in different species and tissue/cell backgrounds, they nevertheless merge in proposing residue Thr116/118 as the central mediator of SOX2/Sox2 protein stability and decay.

According to this model, AKT imposed phosphorylation of Thr116/118 stabilizes SOX2/Sox2 by prevention of its ubiquitination, while AKT-enforced nuclear accumulation of SOX2/Sox2 may be a secondary effect. This distinction may indeed help resolve the uncertainties associated with this site (see paragraph: Phosphorylation of SOX2). Moreover, while K115 may be a target of ubiquitination in human SOX2, this very residue has also been associated with acetylation in murine ESCs (i.e., K117ac), similarly to nearby K119 that has been proposed as a target of Set7 imposed methylation [12, 92]. These findings suggest that acetylation, methylation, phosphorylation, and ubiquitination events imposed onto or nearby the AKT recognition consensus (RPRRK-T116/118) in fact compete with each other.

## PARPylation of SOX2

Sox2 has furthermore been proposed as a target of PARP1 imposed poly(ADP)ribosylation (so-called PARPylation) [118]. In murine ESCs, a competitive association of either PARP1 or Sox2 with the enhancer element of *FGF4*, a gene classically associated with Sox2/Oct4 mediated expression regulation, was described. Furthermore, poly(ADP)ribosylation of Sox2—but not of Oct4—was indicated to causally contribute to its molecular displacement from the enhancer [118]. Although a specific poly(ADP)ribosylation site was not identified, a physical association of SOX2 and PARP1 has been independently confirmed in murine ESCs and human glioma cells [119, 120]. More recently, PARP1 was proposed to assist Sox2 in its target site recognition within densely compacted nucleosomal DNA [121]. These genetically silenced regions are accessible only to so-called pioneer transcription factors, amongst which SOX2 [122], but this cooperativity occurs independent of ADP-ribosylation activity [121].

Accordingly, while a connectivity of SOX2 and PARP1 has been repeatedly observed and appears highly plausible in the context of DNA-activity modulation, and while PARP proteins clearly can transfer poly(ADP) moieties onto neighboring factors, the regulatory significance of SOX2 PARPylation awaits more comprehensive examination.

## O-Glycosylation of SOX2

Finally, SOX2/Sox2 is subject to protein O-glycosylation (O-GlcNAcylation, more specifically) at residue Ser246/248 as shown in the human pancreatic cancer cell line S2VP10, as well as in murine ESCs and iPSCs [119, 123]. Glycosylation at this site decreased with differentiation [123] along with modulation of PARP1 binding [119]. The enzyme imposing this secondary modification has been identified in O-GlcNAc transferase and an inhibition thereof proposed as a novel mode of therapeutic intervention in pancreatic cancer, where small inhibitory compounds of the glycosyl transferase machinery (e.g., OSMI) were shown to suppress tumorigenesis in xenograft models [113]. As with the aforementioned PI3K/AKT imposed phosphor-regulation, protein O-glycosylation also likely represents a recurring molecular theme amongst pluripotency inducing TFs since related modifications were identified in Oct4 and further potential O-GlcNAcylation sites proposed for Sox2 (i.e., Thr258 and Ser259) [123].

## Concluding remarks and perspectives

Taken together, research of the past 25 years near univocally describes SOX2 as a transcriptional modulator that—in association with co-factors—imposes cell-fate determining expression patterns. The molecular understanding of SOX2 nevertheless contains various aspect of effective uncertainty. This involves amongst others how target-site specificity is achieved, relations to the *SOX2* overlapping transcript *SOX2OT*, and finally also, the protein-biochemical fine tuning of SOX2 itself.

Indeed, various secondary modifications have been described for SOX2 (involving phosphorylation, methylation, acetylation, ubiquitination, SUMOylation, PARPylation, and O-GlcNAcylation), but their relevance remains largely underexplored. Identified either in murine or human protein, healthy or transformed cells, native or induced settings, and further obstructed by a somewhat unfortunate residue number shift that distinguishes the otherwise near-identical human and murine orthologues, the various protein-biochemical modifications imposed onto SOX2 were not comprehensively cataloged before.

Our review fills this gap and—as a result of synoptic evaluation—comes to two main conclusions (Fig. 3). Firstly, chemical modification sites described for SOX2 do not randomly scatter throughout the protein’s primary sequence, but cluster in particularly conserved stretches of regulatory significance (i.e., nuclear export sequence (NES), nuclear localization sequence (NLS), and a phosphorylation-dependent SUMOylation motif (PDSM)). Secondly, despite their heterogeneous chemical nature, near all secondary modifications imposed onto SOX2 relate to principally just three functional aspects: DNA-binding, nucleo-cytoplasmatic transport, and protein turnover, which are at the very basis of SOX2 imposed phenotypes. In ignorance of these underlying mechanisms, the biological significance of SOX2 may be described but remains mechanistically unresolved. Even more so as many further but less prominent secondary modifications (e.g., neddylation, urmylation, nitrosylation, or thiolation) have not even been investigated yet, but will complement these concepts with near certainty in the future.

Prospectively, the field would certainly benefit from stringent serial analyses assessing the prevalence of a given secondary modification across multiple cell types on an automated, high-throughput scale. Such endeavor may help assign significance scores to individual site change modifications, define conditions where this regulation is most relevant, and (through the identification of outliers) furthermore indicate compensatory mechanisms. This knowledge could add an important perspective to personalized treatment strategies in patients with SOX2 positive cancers.

Finally, a comprehensive molecular understanding of SOX2 would require a wholistic structural model to enable interpretations of individual covalent side chain modifications in 3-dimensional context, so that steric effects can be assessed and resolved in their functional consequence. Such structural information may guide rational-based drug design against SOX2-driven cancer or improve reprogramming efficacy through molecular fine-tuning of SOX2.
